# Supplementary material for: Targeted Outreach by an Insurance Company Improved Dietary Habits and Urine Sodium/Potassium Ratios Among High-Risk Individuals with Lifestyle-Related Diseases
Source: Nutrients. 2025 Jun 27;17(13):2152. doi: 10.3390/nu17132152 (PMC12252041; doi:10.3390/nu17132152)
Supplement: Supplementary file 1 [file nutrients-17-02152-s001.zip › Figure S1.pdf]

Questionnaires on Health (for customers before intervention)

This questionnaire will be administered to those who have agreed to participate in a joint research project between Hirosaki University and Meiji Yasuda Life Insurance Company (research on the development of a program for education and improvement of pre-symptomatic diseases targeting customers by sales staff of life insurance companies).

We would appreciate your cooperation as we will use this information to provide information and conduct future health-related surveys and research.

The questionnaire is five pages long and takes approximately 5 minutes to complete.

Please ensure that you answer all the questions so that we do not miss any.

The Response results will be processed in a manner that does not identify individuals and will be used solely as statistical survey results.

First Name (Last Name Only) ______________________________

Gender Male / Female

Date of birth (Year/Month/Day) ______________________________

Name of sales staff in charge (last name only) *If unknown, please write "unknown".

____

Q1. Please tell us about yourself

1. Which of the following describes the type of work you do?

☐ Professional and technical occupations ☐ Managerial and admnistrative occupations

☐ Clerical occupations ☐ Sales occupations

☐ Service occupations ☐ Security occupations ☐ Construction and civil engineering occupations

☐ Agriculture ☐ Forestry ☐ Fishing ☐ Transportation and communication worker

☐ Production process and labor worker ☐ Houseworker (housewife, househusband)

☐ Unemployed ☐Student ☐ Unclassifiable

2. Please answer one question that applies to the composition of the household you are currently living with.

☐ A single-person household ☐ Married couple only ☐ Married couple and unmarried children only

☐ A single parent and unmarried children only

☐ Three-generation households (three or more generations)

☐ Households consisting of a couple and their parents ☐ Other households

Q2. Please circle (yes/no) for each of the following items as they apply to your personality.

	Yes	No
① I'm a lively personality.		
② I'm a shy person.		
③ I'm a caring person.		
④ I'm a warm-hearted person.		
⑤ I can't trust even close associates.		
⑥ I don't want to cooperate if it's against me, even if we all agree together.		
⑦ I'm more of a thorough person.		
⑧ I work and study vigorously.		
⑨ I often work on something and stop halfway through.		
⑩ I tend to brood over.		
⑪ Compared to others, I'm a person who can see things for what they are.		
⑫ I'm not good at analyzing problems.		

Q3. Please describe your health situation.

(1) Please provide your current height and weight. **Height: ______ cm** **Weight: ______ kg**

(2) In your most recent health checkup, were there any lifestyle-related diseases requiring attention (including those requiring treatment or close examination)? Please answer one that applies.

☐ No ☐ Yes ☐ I haven't had a physical in a while

(3) (If you answered "Yes" in the above, please indicate all lifestyle-related diseases requiring attention (including those requiring treatment or close examination). (If you answered "other," please specify.

☐ Overweight (obesity) ☐ Hypertension ☐ Diabetes mellitus (positive urine sugar, high blood sugar, high hemoglobin A1c) ☐ Dyslipidemia (high total cholesterol, LDL cholesterol and triglyceride levels, low HDL cholesterol levels)

☐ Hyperuricemia (high uric acid level) ☐ Liver dysfunction (high γ-GTP level, etc.) ☐ Other ( )

(4) Do you currently receive treatment for lifestyle-related diseases, such as medication or regular visits to the hospital? Please answer one that applies.

Lifestyle-related diseases are mainly overweight (obesity), hypertension, diabetes (positive urine sugar, high blood sugar, high hemoglobin A1c), dyslipidemia (high total cholesterol, LDL cholesterol, triglycerides, low HDL cholesterol), hyperuricemia (high uric acid level), and liver dysfunction (high γ-GTP, etc.). GTP levels are high, etc.)

☐ Being treated ☐ Not being treated

(5) Have you had any lifestyle-related diseases pointed out requiring attention (including those requiring treatment or close examination) in your health checkups within the past 5 years?

☐ No ☐ Yes ☐ Have not had a medical checkup in the past 5 years

(6) In the past, have you ever been treated for lifestyle-related diseases by medication or regular visits to the hospital? Please answer one that applies.

Lifestyle-related diseases are mainly overweight (obesity), hypertension, diabetes (positive urine sugar, high blood sugar, high hemoglobin A1c), dyslipidemia (high total cholesterol, LDL cholesterol, triglycerides, low HDL cholesterol), hyperuricemia (high uric acid level), and liver dysfunction (high γ-GTP, etc.). GTP levels are high, etc.)

☐ Had treatment ☐ Had no treatment

(7) Do any of your family members living with you have any illnesses (lifestyle-related diseases) for which you are currently receiving treatment? Please answer one that applies.

Lifestyle-related diseases are mainly overweight (obesity), hypertension, diabetes (positive urine sugar, high blood sugar, high hemoglobin A1c), dyslipidemia (high total cholesterol, LDL cholesterol, triglyceride, low HDL cholesterol), hyperuricemia (high uric acid level), and liver dysfunction (high gamma-GTP value is high, etc.)

☐ No ☐ Yes ☐ Don't know ☐ I'm living solely

Q4. Please answer the following questions about your thoughts on health.

1. What are your current thoughts on improving overall health habits? Please choose one that applies to you.

☐ Not interested in improving overall health habits.

☐ I think we need to improve, but I can't do it.

☐ I want to do something now about improving my overall health habits.

☐ I have already started implementing improvements less than 6 months ago.

☐ I have already started implementing improvements for more than 6 months.

2. Please circle one correct option (1-3) for each of the following items that you think is correct.

About meal	1	2	3
① What is the target daily salt intake for the prevention of hypertension and chronic kidney disease, which is less than how many grams per day?	7.5g	6.5g	6.0g
② What foods are high in soluble fiber that can help prevent high blood pressure and other problems?	avocado	bean curd lees	lettuce
③ The target vegetable of raw intake of 350 g/day is approximately how many areas (cups) of two hands would be equivalent?	2 cups	3 cups	4 cups
About excercise	1	2	3
① Which body parts are particularly prone to loss of muscle mass with aging?	leg	arm	back
② What is your target number of steps per day?<br>* Please answer the number of steps appropriate for your gender.	5,500 steps	9,000 steps	8,500 steps
③ What exercise is particularly effective in addressing the concern of metabolic syndrome/wanting to lower blood pressure?	aerobic exercise	stretch	muscle strength training

3. Please circle the appropriate answer for each of the following items below regarding your current diet and exercise.

	Be applicable	Somewhat applicable	Uundecided	Not very applicable	Not applicable
Dietary bihabior					
① I'm careful not to take too much energy.					
② I'm careful not to eat too much salt.					
③ I'm careful not to take too fat.					
④ I try to consume potassium, vitamins, minerals, and fiber.					
⑤ I try to maintain an appropriate carbohydrate intake.					
motion					
⑥ I perform regular gymnastics/stretching.					
⑦ I’m aware of standing up, moving, standing tall, etc., regularly.					
⑧ I regularly do aerobic exercise (walking, running, and aquabics, etc.)					
⑨ I usually do strength training.					
⑩ I try to incorporate a little exercise into my daily life, such as using the stairs or doing squats during breaks.					

※ Thank you for taking the time to complete our survey.
