# Supplementary material for: Targeted Outreach by an Insurance Company Improved Dietary Habits and Urine Sodium/Potassium Ratios Among High-Risk Individuals with Lifestyle-Related Diseases
Source: Nutrients. 2025 Jun 27;17(13):2152. doi: 10.3390/nu17132152 (PMC12252041; doi:10.3390/nu17132152)
Supplement: Supplementary file 1 [file nutrients-17-02152-s001.zip › Figure S2.pdf]

Figure S2: Health information leaflets provided to the participants of the intervention groups

Towards improving your lifestyle 【Meal】

The content of your diet is closely related to lifestyle-related diseases. The Ministry of Health, Labour and Welfare has established the “Dietary Intake Standards for Japanese” with the aim of maintaining and promoting health and preventing lifestyle-related diseases, so that people can lead a healthy lifestyle. How conscious are you of your diet?

Lifestyle-related diseases linked to diet

Dietary habits	Diseases
Excessive energy intake	Obesity,diabetes,hypertension, ischemic heart disease
Excessive salt intake	High blood pressure, stroke, stomach cancer
Excessive fat intake	Obesity, dyslipidemia, diabetes, ischemic heart disease
Vitamin, mineral, and dietary fiber deficiencies	Various physical ailments such as constipation and anemia

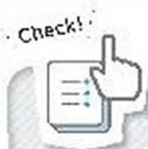

Please pay attention to the points marked with a check mark. Let's focus on learning these points.

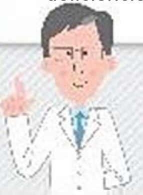

First, let's check your diet!

Answer “Yes” or “No”!

		Date
		Yes /No
1	I sometimes skip a meal a day	
2	I often skip breakfast	
3	I often eat dinner after 9 p.m.	
4	I eat faster than other people	
5	I tend to overeat	
6	I eat single-item meals every day	
7	I often eat out	
8	Snack once or more per day	
9	I eat until full during a snack	
10	I drink sugary soft drinks daily	
11	I drink coffee or tea with sugar daily	
12	I eat sweet breads frequently	
13	I eat fried or greasy foods daily	
14	I eat more meat than fish	
15	I rarely eat seaweed or mushrooms	
16	I eat vegetables in small per day	
17	I rarely eat beans or bean products	
18	I eat ready-to-eat or instant foods	
19	I use mayonnaise or dressing	
20	I drink all the broth from noodle dishes	
21	I add soy sauce or sauce	
22	I drink alcohol daily in excess of moderate amounts	

How many “no” answers were there?

Number of “no” responses

All: Your awareness and habits regarding your diet are excellent.Please continue as you are.16-21: You have a high level of awareness about your diet. Let's keep it up and improve it even further.10-15: Your diet is almost there.Let's try to make a little more effort.
6-9: I know you are busy every day, but why not try to pay a little more attention to your diet? Equal or less than 5: Let's be mindful of lifestyle-related diseases and make improvements where we can. Let's keep up the good work!

Go to Page 2

If you answered “yes” to 8-12, watch your sugar intake.

Go to Page 3

If you answered “yes” to questions 18-21, watch your salt intake.

1 Eat carbohydrates (sugars) in appropriate amout

What is carbohydrates?

Carbohydrates are made up of sugars and dietary fiber. A lack of carbohydrates can cause lethargy, fatigue, and may affect the liver and kidneys.

Carbohydrates

- Sugers
  - Suger
    Monosaccharides and disaccharides
  - Polysaccharides
    A substance consisting of 10 or more monosaccharides linked together.
- Dietary fiber
  The functions of dietary fiber
  1. Suppresses the absorption of cholesterol and glucose in food
  2. Serves as food for good bacteria, regulating the intestinal flora
  3. Increases stool volume, regulating bowel movements

◎Excessive consumption of carbohydrates (sugars) leads to obesity and increases the risk of lifestyle-related diseases.
Carbohydrates are converted into fat when consumed in excess. In particular, sugars, sweets, and fruits raise blood sugar levels rapidly, so be careful not to consume them in excess.

Carbohydrate intake
- Required amount → Energy
- Leftover → Fat

Recommended intake of carbohydrates (sugars): 50–65% of the total calories needed per day for adults

Men: approx. 380 g
Women: approx. 290 g
*For normal physical activity levels

People with high blood sugar levels should adopt lifestyle habits that do not raise blood sugar levels. Those who have been told that their blood sugar levels are high during a health checkup should take caution. Type 2 diabetes, which accounts for 90% of diabetes cases, is closely related to lifestyle habits. By reviewing lifestyle habits such as eating a balanced diet and addressing physical inactivity, prevention and improvement are possible.

Carbohydrate content (approximate) by food item
White rice (regular) 2 cups ・・120g
Shortcake ・・・Approximately 50g
Udon noodles 1 serving ・・・・・80g
Kyoho grapes (1 bunch) ・・48g
Sliced bread 1 slice ・・・・・40g
Daifuku mochi ・・・・・・35g

Foods high in sugar

◯Rice
◯Bread and pasta
◯Soba and udon
◯Potatoes, corn, and pumpkin
◯Fruits
◯Fruit juice
◯Western and Japanese sweets

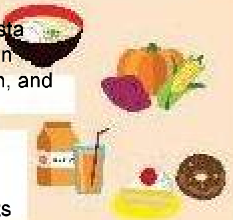

Lifestyle habits that do not raise blood sugar levels

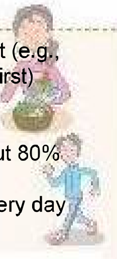

□ Be creative with how you eat (e.g., eat vegetables and seaweed first)
□ Avoid snacking
□ Avoid sugary drinks
□ Aim to eat until you are about 80% full
□ Do light aerobic exercise every day

Let's stabilize blood sugar levels with a regular lifestyle!

2 Let's lower in salt intake

◎How much salt do you consume per day?

The World Health Organization (WHO) has set a stricter target for daily salt intake for adults than Japan, at less than 5g/day, with the aim of preventing high blood pressure and heart disease.

Target sodium intake
Men: Less than 7.5 g
Women: Less than 6.5 g

Stricter target values have been set to prevent the progression of hypertension and chronic kidney disease.
Salt intake target values
Men: Less than 7.5 g
Women: Less than 6.5 g

◎Why is excessive salt intake bad for your health?

High blood pressure has few noticeable symptoms, so if left untreated, it can lead to life-threatening conditions.

Hypertension
↓
Arteriosclerosis
↓
・Cerebral infarction, cerebral hemorrhage, subarachnoid hemorrhage, etc.,Myocardial infarction, angina pectoris, heart failure, Nephrosclerosis, renal failure

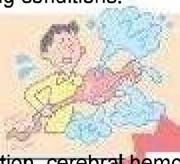

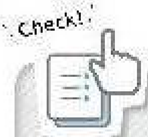

Salt equivalent = "Less than 6.0 g" (for both men and women)

Is your salt intake okay?
Check the applicable items.

Date

- □ Add soy sauce or sauce to flavored side dishes
- □ Drink all the soup from noodles
- □ Add plenty of mayonnaise or dressing
- □ Eat retort pouch or instant foods every day
- □ Eat seaweed or mushrooms about once a week
- □ Don't eat many vegetables

If you checked even one item, please be careful!

Let's find out how many grams of salt are in the foods you usually eat and write it down.

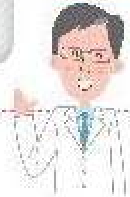

Estimated salt amount included in various foods

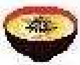	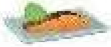	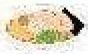	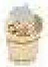	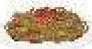	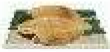
Katsudon	Salted salmon	Ramen	Cup ramen	Sauce yakisoba	Dried hourse mackerel
6.9g	6.5g	5.2g	4.0g	3.8g	2.8g
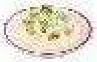	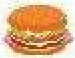	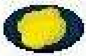	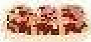	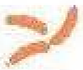	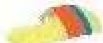
Bongoles spaghetti	Hamburger	Pickled radish	Takoyaki	Wiener sausage	Potato chips
2.6g	2.2g	2.1g	1.9g	1.9g	1.9g

Created based on the Ministry of Education, Culture, Sports, Science and Technology's "Standard Composition of Japanese Foods 2015 Edition (7th Revision)"

3 Recommendations for reducing salt intake and increasing dietary fiber

◎Advice on reducing salt intake
You can reduce your salt intake by making small changes to your diet.
Start by reducing your salt intake little by little and get used to it!

Delicious even with less salt!
A little extra effort in cooking

□ Use fresh ingredients
□ Make the most of the flavor of ingredients
□ Use spices and seasonings
□ Use sour flavors

Advice on reducing salt intake

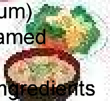

- Eat with vegetables (potassium)
- Salted grilled salmon → Steamed salmon with sake
- Miso soup → Add plenty of ingredients
- Leave half of the ramen broth (leaving half reduces sodium by about 2–3 g)
- Dip instead of pouring soy sauce or sauce
- Low-sodium soy sauce is now available and tastes good

◎Be sure to eat fiber-rich foods
Fiber, which is abundant in vegetables, is believed to be effective in preventing lifestyle-related diseases. The Ministry of Health, Labour and Welfare has set a target intake of 350 grams of vegetables per day and recommends eating this amount every day. Eating plenty of vegetables has the following benefits:

• Potassium helps eliminate excess salt from the body
• Chewing thoroughly helps prevent overeating

The average vegetable intake in Japan falls short of the recommended 350 grams. Please make a conscious effort to increase your vegetable intake.

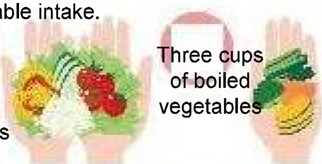

Simply replacing white rice with mixed grains or barley rice will increase your fiber intake.

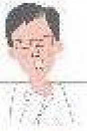

Types and benefits of dietary fiber

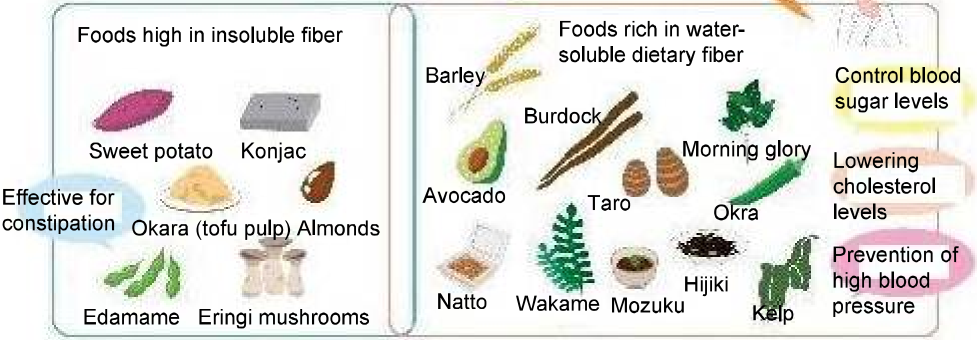

This tool was developed as part of a research project titled “Development of a Preventive Health Education and Improvement Program for Customers Targeted by Sales Staff of Life Insurance Companies” conducted by the COI Pre-disease Science Research Program at Hirosaki University. It will be distributed by sales staff of Meiji Yasuda Life Insurance Company, but it is not intended for use as promotional material.

Toward improving your lifestyle

Exercise habit

According to the Ministry of Health, Labour and Welfare, in Japan, 52,500 people die each year due to physical inactivity. The World Health Organization (WHO) has also reported that physical inactivity is the fourth leading risk factor for global mortality, following hypertension, smoking, and high blood sugar. Recent surveys indicate that Japanese people spend more time sitting than anyone else in the world. In particular, office workers tend to spend long periods of time sitting, so it is important to consciously move your body at least once an hour, such as standing up, stretching, or walking around.

Effects of exercise

① Improve muscle strength
② Improve cardiorespiratory function and blood circulation
③ Helpful for weight loss and maintaining body shape
④ Helpful for stress relief
⑤ Helpful for preventing various diseases such as osteoporosis, diabetes, arteriosclerosis, etc.

Check!

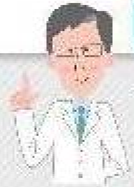

Are you getting enough exercise?

First, let's check your activity level! Are you getting enough exercise? Let's check the table below.

Date:

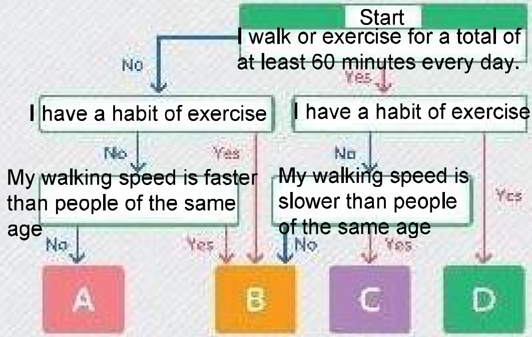

Your type is

A If you continue like this, we are concerned about your health.

B Just a little more to go until you reach your goal!

C You are achieving our goals.

D Great! Please get more people to join us and exercise together!

We recommend you to measure your waist circumference

"Lack of exercise" and "overeating" affect waist circumference.
As a review of your lifestyle, let's measure your waist circumference.
The standard values are 85 cm for men and 90 cm for women. If you are above or below these values and meet two or more of the following three criteria, you are at risk for metabolic syndrome!

Blood pressure: Systolic 130 mmHg or higher or diastolic 85 mmHg or higher
Blood sugar: Fasting blood sugar 110 mg/dl or higher
Dyslipidemia: Triglycerides 150 mg/dl or higher or HDL cholesterol less than 40 mg

1 Does aging start from the feet?

Muscle mass is said to decline with age from the 30s to 40s, and by the 70s, it is reduced by about half. Leg muscles are particularly prone to decline, and this is related to how often they are used on a daily basis. Recent studies have shown that even people over the age of 60 can maintain or increase muscle mass through training that puts stress on the muscles.

◎ Do you know 'locomotive syndrome' ?

Locomotive syndrome refers to a condition in which disorders of the musculoskeletal system (joints, bones, muscles, etc.) cause a decline in walking ability, muscle strength, balance, and other functions, thereby affecting daily life. If left untreated, it can impact healthy life expectancy and quality of life. However, like lifestyle-related diseases, it is possible to restore the body to a state close to its original condition by improving diet and exercise habits.

Are your habits okay?

- □ No exercise habits
- □ Underweight/overweight
- □ Low activity level
- □ Long periods of sitting
- □ Excessive strenuous exercise
- □ Ignoring lower back or knee pain
- □ Ignoring injuries

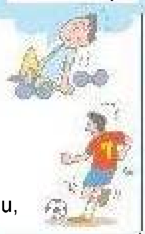

If you think this applies to you, take a look at your lifestyle.

Exercise for Health (3 Essential Elements)

Aerobic exercise
For Cardiopulmonary function, suppression of arteriosclerosis, and improvement of sugar and lipid metabolism

Strength training
Improve activity, and prevention of falls

Stretching
Improvement of activity and prevention of disability

Let's try the stand-up test!

Assessment of locomotive syndrome
[level 1] Unable to stand up from a height of 40 cm on one leg
[level 2] Unable to stand up from a height of 20 cm on both legs

If you experience pain when standing up with both feet
Consult a medical professional.

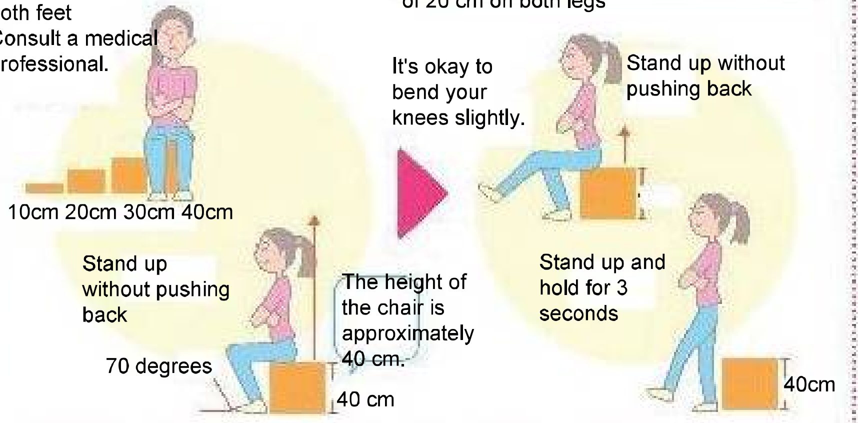

2 Healthy habits through walking

Walking is an aerobic exercise that anyone can do. You probably walk for a few minutes to a few tens of minutes every day. The key to increasing the amount of exercise is to consciously change your unconscious actions. Simply switching from “walking” to “walking consciously” will lead to more effective walking.

《Start walking to combat metabolic syndrome and locomotive syndrome!》

Target value (Healthy Japan 21)	Tips for increasing your step count
Men: 9,200 steps/day<br>Women: 8,300 steps/day	1. Walk instead of driving<br>2. Use stairs instead of elevators

The benefits of proper walking

- □ Become less prone to weight gain
- □ Increase muscle strength in the abdominal and back muscles
- □ Improve posture and sitting posture
- □ Improve bowel movements and reduce acne
- □ Reduce shoulder stiffness, lower back pain, and knee pain
- □ Reduce the risk of dementia
- □ Reduce the risk of heart disease, arteriosclerosis, and cancer
- □ Activate metabolism and look younger

STEP 1 First, reduce the amount of time you spend sitting.

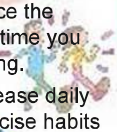

STEP 2 Increase daily exercise habits

STEP 3 Do some light exercise

First, try walking for 10 minutes longer than you normally do, using the correct walking technique!

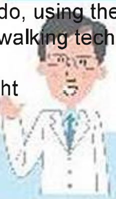

How to walk correctly

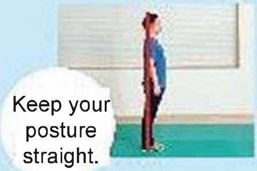

Keep your posture straight.

Align your ears, shoulders, hips, knees, and ankles in a straight line.Stand up straight, keeping this in mind.

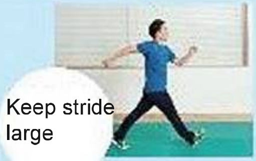

Keep stride large

Walk with as large a stride as possible. A good guideline is “your height minus 100 cm.”

Additional advice

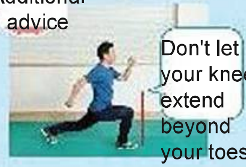

Don't let your knees extend beyond your toes.

Walking with long strides improves leg strength and hip flexibility!

(Always keep in mind the key points of effective walking.)

Shifting your center of gravity from your heels to your toes

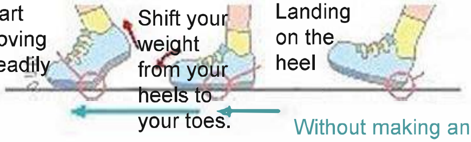

Start moving steadily

Shift your weight from your heels to your toes.

Landing on the heel

Without making any noise, roll it along.

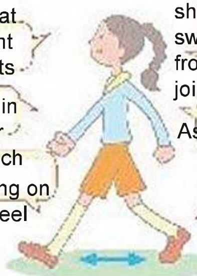

Look at distant objects

Suck in your stomach

landing on the heel

Relax your shoulders and swing your arms from the shoulder joints.

As if stretching

Finally, kick firmly.

3 Let's move more 10 minutes in our daily lives.

The Ministry of Health, Labour and Welfare recommends increasing physical activity by 10 minutes per day in its “Physical Activity Guidelines for Health Promotion.”

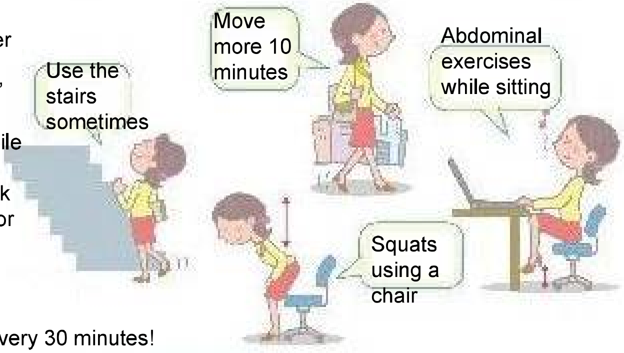

Physical activities you can do while commuting or at work

- Get off one stop earlier and walk
- Use stairs instead of elevators or escalators at stations and workplaces
- Commute by bicycle

Stand up from your chair once every 30 minutes!

Recommended exercises for specific concerns

Stiff shoulders / Fatigue / Neck pain / Irritability, etc.	Stretching; Yoga and flexibility exercises are recommended.
Walking or running is painful / My legs and hips have become weak... etc.	Let's do some strength training Abdominal exercises and squats are recommended.
Concerned about metabolic syndrome/ Want to lower blood pressure...etc.	Let's do aerobic exercise Walking and aqua aerobics are recommended.

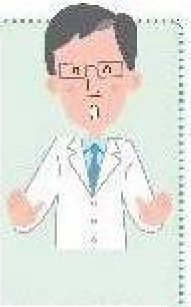

“+10” in walking is +1,000 steps!

The guideline for “+10” walking is 1,000 steps or 700 meters.

◆Even your everyday shopping can add up to “+10”! When you shop at a major shopping center in Aomori City, you can walk an average of about 10 minutes and take about 790 steps.

◆By shopping frequently for what you need when you need it, you can add up to “+10”.

◆Try using a pedometer or walking app to measure and record your daily steps and exercise!

This tool was created as part of a study conducted by the COI Mibyo Science Research Program at Hirosaki University on “Development of a mibyo education and improvement program for customers targeted by life insurance company sales staff.” It will be distributed by Meiji Yasuda Life Insurance Company sales staff, but it is not intended to be used as promotional material.

*This study is a continuation of research conducted in 2019 to examine more effective health education intervention methods by sales staff. It involves interventions based on personality types and health awareness and conditions, and verifies their effectiveness.
