# Supplementary material for: Targeted Outreach by an Insurance Company Improved Dietary Habits and Urine Sodium/Potassium Ratios Among High-Risk Individuals with Lifestyle-Related Diseases
Source: Nutrients. 2025 Jun 27;17(13):2152. doi: 10.3390/nu17132152 (PMC12252041; doi:10.3390/nu17132152)
Supplement: Supplementary file 1 [file nutrients-17-02152-s001.zip › Figure S3.pdf]

Figure S3. The post-interventional questionnaire (translated into English for publication)

Questionnaires on Health (for customers after intervention)

This questionnaire will be administered to those who have agreed to participate in a joint research project between Hirosaki University and Meiji Yasuda Life Insurance Company (research on the development of a program for education and improvement of pre-symptomatic diseases targeting customers by sales staff of life insurance companies).

We would appreciate your cooperation as we will use this information to provide information and conduct future health-related surveys and research.

The questionnaire is five pages long and takes approximately 5 minutes to complete.

Please ensure that you answer all the questions so that we do not miss any.

The Response results will be processed in a manner that does not identify individuals and will be used solely as statistical survey results.

First Name (Last Name Only) ______________________

Gender Male / Female

Date of birth (Year/Month/Day) ______________________

Name of sales staff in charge (last name only) *If unknown, please write "unknown".

____

Q1. Please provide your current height and weight

(Considering the purpose of this study, please indicate your height and weight as of today so that we can compare your weight before and after.)

Please provide your current height and weight. Height: cm Weight: kg

Q2. Please circle one correct option (1-3) for each of the following items that you think is correct.

About meal	1	2	3
① What is the target daily salt intake for the prevention of hypertension and chronic kidney disease, which is less than how many grams per day?	7.5g	6.5g	6.0g
② What foods are high in soluble fiber that can help prevent high blood pressure and other problems?	avocado	bean curd lees	lettuce
③ The target vegetable of raw intake of 350 g/day is approximately how many areas (cups) of two hands would be equivalent?	2 cups	3 cups	4 cups
About excercise	1	2	3
① Which body parts are particularly prone to loss of muscle mass with aging?	leg	arm	back

②	What is your target number of steps per day?<br>* Please answer the number of steps appropriate for your gender.	5,500 steps	9,000 steps	8,500 steps
③	What exercise is particularly effective in addressing the concern of metabolic syndrome/wanting to lower blood pressure?	aerobic exercise	stretch	muscle strength training

Q3. Please circle the appropriate answer for each of the following items below regarding your current diet and exercise.

	Be applicable	Somewhat applicable	Undecided	Not very applicable	Not applicable
Dietary behavior					
① I'm careful not to take too much energy.					
② I'm careful not to eat too much salt.					
③ I'm careful not to take too fat.					
④ I try to consume potassium, vitamins, minerals, and fiber.					
⑤ I try to maintain an appropriate carbohydrate intake.					
Locomotor activities					
⑥ I perform regular gymnastics/stretching.					
⑦ I’m aware of standing up, moving, standing tall, etc., regularly.					
⑧ I regularly do aerobic exercise (walking, running, and aquabics, etc.)					
⑨ I usually do strength training.					
⑩ I try to incorporate a little exercise into my daily life, such as using the stairs or doing squats during breaks.					

Q4. This time, the Meiji Yasuda Life Insurance's sales staff offered three different health behavior change initiatives. Please answer all the questions that left a strong impression on you.

☐ Information and use of the urinary analysis (salt intake test)

☐ Providing health information leaflets

☐ Courtesy of a health-related video

☐ Not applicable for response (did not receive health behavior change encouragement from sales staff)

Please answer the reason for Q4.

(   )

Q5. Did you read the health information leaflets presented by the Meiji Yasuda Life Insurance sales staff this time? Please choose one that applies to you.

☐ I've generally read it.

☐ I read only part of it (about _______%).

☐ I haven't read it at all.

☐ Not applicable for response (did not receive health behavior change encouragement from sales staff)

Q6. Did you watch the health-related videos introduced by Meiji Yasuda Life Insurance sales staff this time? Please choose one that applies to you.

☐ I generally saw it.

☐ I saw only part of it (about _______%)

☐ I haven't seen it at all.

☐ Not applicable for response (did not receive health behavior change encouragement from sales staff)

Q7. This time, you used the MY Hoken app to measure your steps. Did you remember to carry your smartphone with you when you went out? (If you sometimes forgot your smartphone, please indicate how many percent of your outings during the period you carried your smartphone with you)

☐ I generally carried my phone with me when I went out.

☐ Sometimes I forgot my phone and went out. (I carried my phone with me for about _______% of the time during the period.)

Q8. In the past month or two, do you think your awareness of your overall health has increased? Please choose one that applies to you.

☐ Increased considerably ☐ Increased somewhat ☐ Can't say either

☐ Not very much ☐ Not at all

Q9. In the past month or two, have you started or resumed treatment for a lifestyle-related disease? Please choose one that applies to you.

☐ I started treatment for lifestyle-related diseases.

☐ I Resumed treatment for lifestyle-related diseases.

☐ No treatment for lifestyle-related diseases has been done so far.

Q10. Did the urinalysis service (salt intake test) provided by Meiji Yasuda Life Insurance sales staff influence your health consciousness? Please choose one that applies to you.

☐ Fairly affected ☐ Somewhat affected ☐ Can't say either

☐ Not much affected ☐ Not at all affected

Q11. Did the health information leaflets provided by Meiji Yasuda Life Insurance sales staff influence your health awareness? Please choose one that applies to you.

☐ Fairly affected ☐ Somewhat affected ☐ Can't say either

☐ Not much affected ☐ Not at all affected

☐ Not applicable for response (did not receive health behavior change encouragement from sales staff)

Q12. Did the health-related videos provided by Meiji Yasuda Life Insurance sales staff influence your health awareness? Please choose one that applies to you.

☐ Fairly affected ☐ Somewhat affected ☐ Can't say either

☐ Not much affected ☐ Not at all affected

☐ Not applicable for response (did not receive health behavior change encouragement from sales staff)

Q13. How do you think about improving your overall health habits in the future? Please choose one that applies to you.

☐ Not interested in improving overall health habits.

☐ I think we need to improve, but I can't do it.

☐ I want to do something now about improving my overall health habits.

☐ In the past month or two, I have begun to act on improving our overall health habits.

☐ I have already started implementing improvements less than 3 to 6 months ago.

☐ I have already started implementing improvements for more than 6 months.

Q14. We would appreciate your feedback on your participation in this health-related study.

Thank you very much for your cooperation in completing the survey.
