# Supplementary material for: Targeted Outreach by an Insurance Company Improved Dietary Habits and Urine Sodium/Potassium Ratios Among High-Risk Individuals with Lifestyle-Related Diseases
Source: Nutrients. 2025 Jun 27;17(13):2152. doi: 10.3390/nu17132152 (PMC12252041; doi:10.3390/nu17132152)
Supplement: Supplementary file 1 [file nutrients-17-02152-s001.zip › Table S1.pdf]

Table S1. The algorithm for determining a client's "Cash-back rank"

Step 1. Each item of the Specific Health Check-up results is categorized as A to D.

	A	B	C	D
BMI	18.5 – 24.9	15.0 – 18.4, 25.0 - 29.9	30.0 – 34.9	≦ 14.9, ≧ 35.0
SBP (mmHg)	≦ 129	130 – 139	140 – 159	≧ 160
DBP (mmHg)	≦ 84	85 – 89	90 – 99	≧ 100
Qualitative Glucosuria† (test strip method)	(-)	(±) , (+), (+ +), (+ + +)		
Qualitative proteinuria (test strip method)	(-)	(±)	(+)	(+ +), (+ + +)
Triglycerides‡ (mg/dl)	30 - 149	150 – 299	300 -499	≧ 500
ALT (U/L)	≦ 30	31 -40	41 – 50	≧ 51
γ- GTP (U/L)	≦ 50	51 -80	81 – 100	≧ 101
HbA1c‡ (%)	≦ 5.5	5.6 – 5.9	6.0 – 6.4	≧ 6.5
FBS (mg/dL)	≦ 99	100 - 109	110 -125	≧ 126

BMI: body mass index, SBP: systolic blood pressure, DBP: diastolic blood pressure, AST: Aspartate aminotransferase, γ-GTP: gamma-glutamyltransferase, HbA1c: hemoglobin A1c, FBS: fasting blood sugar. †Applied for < 40 years old, ‡Applied for ≧ 40 years old.

Step 2. 'Cash-back points' are given for each item based on the category

1) Under 40 years old

	A	B	C	D
BMI	30	20	Male: 0<br>Female: 10	0
Blood pressure (SBP or DBP)	30	Male: 20<br>Female: 10	Male: 10<br>Female: 0	0
Glucosuria	30	0		
Proteinuria	30	20	Male: 0<br>Female: 10	0

Triglycerides	10	0
ALT or γ-GTP		
FBS (mg/dL)		

BMI: body mass index, SBP: systolic blood pressure, DBP: diastolic blood pressure, AST: Aspartate aminotransferase, γ-GTP: gamma-glutamyltransferase, FBS: fasting blood sugar.

2) Forty years old or older

	A	B	C	D
BMI	30	Male: 20<br>Female:10	Male: 10<br>Female: 0	0
Blood pressure (SBP or DBP)	30	20	10	0
Proteinuria	30	20	0	0
Triglycerides	30	Male: 20<br>Female: 10	Male:10<br>Female: 0	0
AST or γ-GTP	30	Male: 20<br>Female: 10	Male: 10<br>Female: 0	0
HbA1c (%) or FBS (mg/dL)	30	Male: 10<br>Female: 20	0	0

BMI: body mass index, SBP: systolic blood pressure, DBP: diastolic blood pressure, AST: Aspartate aminotransferase, γ-GTP: gamma-glutamyltransferase, HbA1c: hemoglobin A1c, FBS: fasting blood sugar.

Step 3. Determination of 'Cash-back rank' based on the total 'Cash-back points'

	< 40 years old			≧ 40 years old		
'Cash-back rank'	1	2	3	1	2	3
Total 'Cash-back points'	≧ 120	110	≦ 100	≧ 170	150 - 160	≦ 140
